# Genetic link between depression and musculoskeletal disorders: insights from Mendelian randomization analysis

**DOI:** 10.3389/fmed.2024.1398203

**Published:** 2024-05-31

**Authors:** Yanpeng Wang, Yinzhen Zhang, Changwei Zhao, Hao Yang, Chenglong Ai, Wenhai Zhao, Ji Xu

**Affiliations:** ^1^Department of Traditional Chinese Medicine, Changchun University of Chinese Medicine, Changchun, China; ^2^Department of Orthopedics, Affiliated Hospital of Changchun University of Chinese Medicine, Changchun, China; ^3^Department of Spinal Orthopedics, Weifang Hospital of Traditional Chinese Medicine, Shandong Second Medical University, Weifang, China

**Keywords:** depression, cervical spondylosis, musculoskeletal diseases, Mendelian randomization, GWAS

## Abstract

**Background:**

The association between depression and musculoskeletal diseases has long been a subject of contentious debate. However, the causal relationship between the two remains uncertain. This study employs a two-sample Mendelian randomization (MR) analysis to investigate the causality between depression and six musculoskeletal diseases.

**Methods:**

In this study, we performed MR analysis to systematically explore the causal relationship between depression and six musculoskeletal disorders. Single nucleotide polymorphisms (SNPs) that are linked to depression were employed as instrumental variables. To ensure robust and reliable conclusions, multiple analytical approaches were utilized, including inverse variance weighting(IVW), weighted median, and MR-Egger regression. Additionally, sensitivity analysis methods such as the MR-Egger intercept test, Cochran’s Q test, leave-one-out analysis, and funnel plot were employed.

**Results:**

Our MR analysis revealed a significant association between depression and cervical spondylosis (depression: OR 1.003, 95% CI 1.002–1.005, *P* = 8.32E-05; major depressive disorder: OR 1.003, 95% CI 1.001–1.005, *P* = 0.0052). Furthermore, a strong correlation was noted between major depressive disorder (MDD) and knee osteoarthritis (KOA) (OR 1.299, 95% CI 1.154–1.463, *P* = 1.50E−5). Sensitivity analysis confirmed the robustness of these findings. Our independent validation study also corroborated these results.

**Conclusion:**

The MR analysis conducted in this study provides evidence supporting a genetic link between depression and cervical spondylosis, as well as KOA. Targeted interventions to manage depression in susceptible populations may contribute to lowering the risk of cervical spondylosis and KOA in these cohorts.

## 1 Introduction

Depression is a prevalent, debilitating, and costly mental disorder worldwide, affecting more than 300 million individuals. MDD represents a severe form of depression, presenting significant challenges in terms of treatment and profoundly impacting patients’ emotional, physical well-being, daily functioning, learning, and work (1, 2). Previous research has not only demonstrated its adverse effects on cardiovascular and metabolic diseases (3), but also indicated its significant role in specific postural changes in the human body (4), highlighting the close relationship between depression and the occurrence of musculoskeletal diseases (5, 6).

Musculoskeletal diseases (MSD) primarily include chronic pain-related conditions such as cervical spondylosis (CS), KOA, hip osteoarthritis (HOA), rheumatoid arthritis (RA), lumbar disc herniation (LDH), and osteoporosis (OP). These conditions typically manifest as chronic pain and restricted mobility, imposing substantial economic and societal burdens (7), while profoundly impacting individuals’ quality of life (8).Previous observational studies have indicated that CS patients face an elevated risk of depression (9), with similar trends observed in KOA (10), RA (11) and OP patients. However, limited by experimental design and sample size, few studies have explored whether depression increases the risk of MSD. Observational studies are inherently limited by confounding factors and reverse causality, hindering the determination of associations at a genetic level. Furthermore, comprehensive research on the relationship between depression and MSD remains lacking. MR offers a new perspective to address these challenges.

MR is a precise medical analytical method that utilizes genetic variations to establish associations between risk factors and outcomes, thereby evaluating causal effects (12). By capitalizing on the random assignment of genetic variations during conception, MR analysis introduces randomness and mitigates potential confounding factors and reverse causality issues commonly encountered in observational studies. This method effectively integrates genetic information into traditional epidemiological investigations, offering a fresh approach to elucidate the associations between different factors. Therefore, we posit that utilizing the MR approach aids in comprehending the causal relationship between depression and MSD. This study aims to employ MR analysis to investigate the potential association between depression and MSD, thereby offering valuable insights for both preventive and therapeutic interventions.

## 2 Method and design

### 2.1 Research design

**FIGURE 1:**
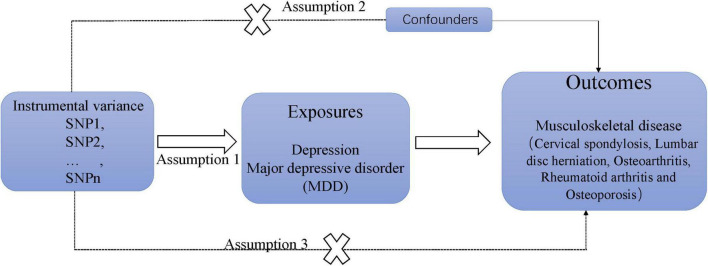
Basic hypothesis of mendelian randomization.

Figure 1 illustrates the research design and the hypothesis regarding MR utilized in this study. The study employed a two-sample MR analysis and aggregated data from genome-wide association studies (GWAS) to investigate the causal effects of depression on chronic sinusitis CS, KOA, HOA, RA, LDH, and OP. Additionally, independent verification tests were conducted to ensure the reliability of the results. The MR design is guided by three key assumptions: (1) instrumental variables demonstrate a strong correlation with depression; (2) the instrumental variable is not linked to confounding factors; and (3) the association with MSD risk is solely mediated through depression (13).

### 2.2 Data sources

The depression phenotypes were categorized as depression and MDD. Data on depression included 23,424 patients and 192,220 controls from the Fingen, while data on MDD comprised 170,756 patients and 329,443 controls from the IEU Open GWAS. Furthermore, self-reported datasets on CS with 3,292 patients and 459,641 controls, and LDH with 2,214 patients and 460,796 controls, were obtained through a meta-analysis of UK Biobank sources in GWAS. The datasets on KOA with 24,955 patients and 378,169 controls, HOA with 15,704 patients and 378,169 controls, RA with 14,361 patients and 43,923 controls, and OP with 3,203 patients and 209,575 controls were all sourced from the IEU Open GWAS. In the validation cohort, CS data were extracted from the IEU Open GWAS, encompassing 3,292 cases and 459,641 controls, while KOA data were obtained from the Genetics of Osteoarthritis (GO) (14) Consortium, comprising 62,497 cases and 333,557 controls. All genetic data included in this study were sourced from patients of European descent and were accessible from the open GWAS dataset.^1^ Since the data used in this study are derived from existing publications and public databases, no additional ethical approval or consent is required for participation.

### 2.3 Selection of instrumental variables

First, we select SNP at the genome-wide significant level (*P* < 5 × 10^–8^) (since only one SNP is associated with CS at the genome-wide significant level, so we set the threshold to the significant level *p* < 5 × 10^–6^); then, to remove the linkage disequilibrium, set the genetic distance to 10000kb, and set the linkage disequilibrium parameter r^2^ threshold = 0.001; third, remove ambiguity and palindromes SNP through the coordination process. Fourth, F statistics are used to evaluate whether the included SNPs is affected by weak tool variables, and the corresponding SNP of *F* < 10 is eliminated. Finally, we removed SNPs associated with BMI, obesity, lipids, and outcomes through online websites Phenocsnner.

### 2.4 Statistical analysis

In our study, we applied the IVW method with random effects as the primary analytical approach. Moreover, we employed the Weighted Median and MR Egger methods as supplementary techniques to the IVW method. The IVW method yields precise estimates under the assumption that all SNPs serve as valid instrumental variables (15). To assess estimates under the presence of directional pleiotropy, we utilized the MR Egger and Weighted Median methods, which offer increased robustness (16, 17). These methods were used for comparative analysis in conjunction with the IVW estimates. The MR-PRESSO method was employed in this study to detect any outliers, and any outliers identified were removed for reanalysis. The results were presented as odds ratios (ORs) with their corresponding 95% confidence intervals (CIs), providing an estimate of the associated risk following a one-standard deviation (SD) increase in the risk factors.

We have observed a considerable degree of sample overlap between MDD and the outcome variable. Previous MR analyses (18, 19) have indicated that, in two-sample MR studies, aside from the MR-Egger method, other methodologies can confidently employ the same biological sample repository for analysis purposes. Given our utilization of the IVW method as the primary analytical approach, our findings can to some degree discount the bias stemming from sample overlap.

### 2.5 Sensitivity testing

The quality control of the results calculated by MR was carried out by sensitivity test. It mainly includes heterogeneity test and pleiotropic test. In addition, in order to prevent bias in the analysis results, this study also conducted a ‘leave-one-out’ and funnel plot sensitivity test.

The analysis in this study was conducted using R4.2.1 software and the Two sample MR package. Following Bonferroni correction (0.05/6), statistical significance was determined at *P* < 0.008. Values between 0.05 and 0.08 were deemed nominally significant.

## 3 Results

### 3.1 Instrumental variables

Following screening, we initially incorporated 30 SNP instrumental variables for depression and 50 SNP instrumental variables for MDD. Subsequently removing repetitive and palindromic sequences (rs12491634, rs2876520, rs4730387, rs35469634), we ultimately opted for 29 and 47 SNPs as instrumental variables to estimate depression and MDD, respectively (Supplementary Table 1).

The instrumental variables utilized in this study accounted for approximately 4.1% and 1.7% of the genetic variance of depression and MDD, respectively. Importantly, the F values associated with the selected SNPs exceeded 10, thereby ruling out the presence of weak instrumental variables (Supplementary Table 2).

### 3.2 MR analysis of depression and MDD on MSD

**FIGURE 2:**
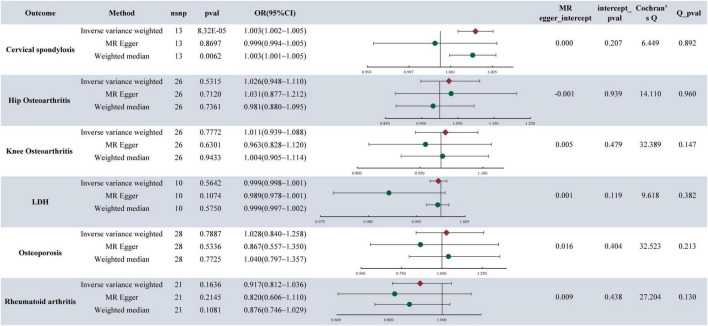
MR analysis and sensitivity analysis results of depression and six musculoskeletal diseases.

**FIGURE 3:**
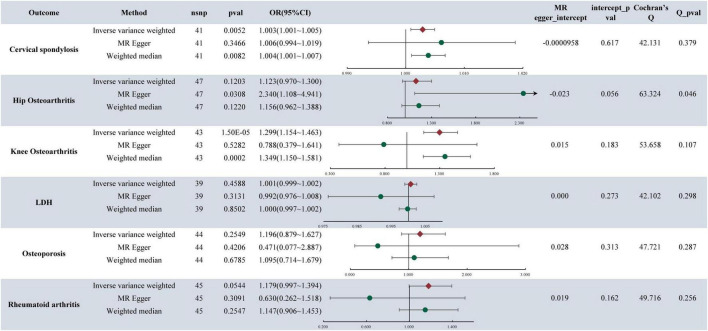
MR analysis and sensitivity analysis results of major depressive disorder and six musculoskeletal diseases.

In the analysis, it was found that depression is associated with an increased likelihood of CS (OR = 1.003, 95%CI: 1.002–1.005, *P*-value = 8.32E**−**05) (refer to Figure 2). Similarly, MDD also elevates the risk of CS, with an OR of 1.003 (95% CI: 1.001–1.005, *P*-value = 0.0052). Furthermore, the research indicated that MDD is linked to a higher risk of KOA, with an OR of 1.299 (95% CI: 1.154–1.463, *P*-value = 1.50E**−**5). Notably, no causal relationship was found between depression, MDD, and HOA,LDH, RA, OP. For detailed results, please refer to Figure 3.

### 3.3 Sensitivity analysis results

The presence of pleiotropy in our study was assessed through the examination of the MR-Egger regression intercept. The MR Egger intercept values for depression, MDD, and CS were 0.0003 and **−**0.0001, respectively, indicating values close to 0. Both corresponding *p*-values, 0.2075 and 0.6167, exceeded 0.05. These results imply the absence of horizontal pleiotropy and multiple effects interference in the MR analysis. Conversely, the MR Egger intercept values for MDD and KOA were 0.015 and 0.183, both exceeding 0.05, suggesting no presence of horizontal pleiotropy (Figures 2, 3).

The heterogeneity tests were conducted to evaluate the variability within the dataset. For depression, MDD, and CS, the Cochran’s Q values and QP values for the IVW method were 6.4495 and 42.1315, respectively, both surpassing the significance threshold of 0.05. These findings indicate the absence of heterogeneity and confirm the reliability of the results. Similarly, in patients with MDD and KOA, the Cochran’s Q and QP values for IVW were 53.658 and 0.107, respectively, exceeding 0.05, suggesting no heterogeneity in the data (refer to Figures 2, 3).

Sensitivity analyses were conducted using the “Leave-one-out” method and funnel plots. These analyses did not reveal any significant abnormalities, as illustrated in Supplementary Figures 1–3.

### 3.4 Validation cohort

**FIGURE 4:**
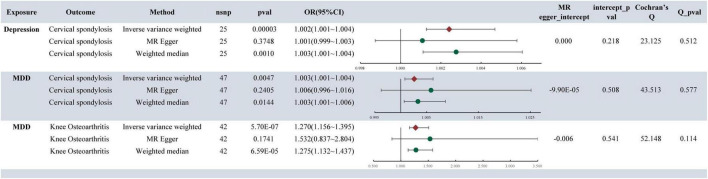
MR analysis and sensitivity analysis results of validation cohort.

In our re-analysis of the validation cohorts for CS and KOA, consistent trends persist. The findings indicated a continued significant association between depression and CS at the genetic level [depression: (OR: 1.002, 95% CI: 1.001–1.004, *p* = 0.00003)], MDD [OR: 1.003, 95% CI: 1.001–1.004, *p* = 0.0047)], along with analogous results observed between MDP and KOA (OR: 1.270, 95% CI: 1.156–1.395, *p* = 5.7E**−**07). Furthermore, sensitivity analysis confirmed the robustness of the findings (Figure 4).

## 4 Discussion

In this study, the GWAS dataset was utilized to thoroughly investigate the causal relationship between depression and six musculoskeletal diseases. The findings revealed a genetic-level association between depression and CS, albeit with a small effect size. Furthermore, our research indicated a link between depression and the susceptibility to developing KOA. Conversely, no causal relationship was found between depression, MDD, and other conditions such as HOA, LDH,RA, or OP.

Previous observational studies have reported an elevated risk of depression in patients with CS (20, 21). However, limited research exists on whether depression contributes to an increased risk of CS. A cohort study involving 68,000 individuals in Taiwan, utilizing data from the Taiwan Health Insurance Research Database, demonstrated a 76% higher risk of CS in individuals with depression compared to those without depression; notably, the use of specific antidepressant medications did not mitigate this risk (22). These findings align with our study results. Regarding the relationship between depression and knee pain, well-conducted studies (23) have indicated that depression heightens the risk of knee pain in adults, and antidepressant use is linked to relieving knee pain. Nevertheless, limited observational data exist on whether depression increases the likelihood of KOA. Hence, further validation through extensive cohort studies is essential.

The increased risk of CS and KOA among individuals with depression may be closely linked to poor posture. Research indicates that individuals with depression often exhibit poor posture, which can lead to neck muscle tension and alterations in spinal structure, potentially contributing to the development of CS (4–6). Moreover, individuals with depression often engage in decreased physical activity, leading to muscle atrophy, reduced joint stability, and potentially adverse effects on knee function and outcomes (24).

In exploring the underlying mechanism of the causal relationship between depression and MSD, pro-inflammatory factors are significantly implicated. Prior studies have established a positive connection between inflammatory markers and MDD (25–29), including IL-6, IL-1β, CRP, and TNF. The heightened levels of these inflammatory markers are also prominent drivers of CS by contributing to degeneration and disc deterioration in the cervical region (30). Yin et al. (31) have underscored that these inflammatory factors may incite intervertebral disc degeneration and neuropathic pain, ultimately culminating in the development of CS (32). Additionally, research has delineated a strong relationship between the inflammatory process and the pathophysiology of depression, indicating that inflammation may serve as a critical disease-modifying factor that increases susceptibility to depression (33). Furthermore, evidence suggests that medications prescribed for depression could potentially foster the development of CS. Lin et al. (22) conducted an investigation involving 34,166 patients with depression, segregating them into two groups: one treated with antidepressants and the other without. The outcomes indicated that the incidence of CS in the medication group was 1.8 times higher in comparison to the non-medication group.

The primary strength of our study lies in being the first MR investigation to comprehensively examine the association between depression and MSD. By leveraging the inherent strengths of MR methodology, this study minimizes biases arising from confounding variables and reverse causation. The findings also highlight the impact of depression on the genetic susceptibility to CS and KOA. Nonetheless, several limitations warrant acknowledgment. Firstly, the majority of participants in the MR analysis are of European descent, potentially introducing racial bias concerns. Secondly, due to constraints in the available data, a more thorough subgroup analysis assessing the influence of MDD on MSD outcomes was not feasible. Additionally, it is pertinent to investigate the possible causal relationships between inflammatory mediators and both CS and depression.

## 5 Conclusion

The MR analysis conducted in our study reveals a genetic link between depression and both CS and KOA. Improving the care and support for populations vulnerable to depression could potentially mitigate the incidence of MSD in these specific cohorts.

## Data availability statement

The original contributions presented in this study are included in the article/Supplementary material, further inquiries can be directed to the corresponding author.

## Author contributions

YW: Conceptualization, Writing – original draft, Writing – review and editing. YZ: Conceptualization, Writing – original draft. CZ: Data curation, Investigation, Writing – review and editing. HY: Data curation, Software, Writing – review and editing. CA: Data curation, Software, Writing – review and editing. WZ: Funding acquisition, Supervision, Validation, Visualization, Writing – review and editing. JX: Funding acquisition, Supervision, Validation, Visualization, Writing – review and editing.

## Footnotes

1
https://gwas.mrcieu.ac.uk
