# Supplementary material for: Genetic link between depression and musculoskeletal disorders: insights from Mendelian randomization analysis
Source: Front Med (Lausanne). 2024 May 31;11:1398203. doi: 10.3389/fmed.2024.1398203 (PMC11177873; doi:10.3389/fmed.2024.1398203)
Supplement: Supplementary file 1 [file Table_1.docx]

Supplementary Material

**Table 1**, Basic information of included instrumental variable

| **Exposure** | **SNP** | **EA** | **OEA** | **BETA** | **EAF** | **SE** | **P-value** |
| --- | --- | --- | --- | --- | --- | --- | --- |
| Depression | rs10058289 | G | A | 0.0536 | 0.4311 | 0.0111 | 1.25E-06 |
| Depression | rs10140802 | T | C | -0.052 | 0.6278 | 0.0112 | 3.72E-06 |
| Depression | rs10880262 | T | C | -0.0518 | 0.5076 | 0.0109 | 2.10E-06 |
| Depression | rs111399861 | T | G | -0.1356 | 0.0405 | 0.0293 | 3.54E-06 |
| Depression | rs11159917 | T | C | 0.0599 | 0.2444 | 0.0126 | 2.13E-06 |
| Depression | rs113392839 | A | G | 0.3299 | 0.007743 | 0.0632 | 1.76E-07 |
| Depression | rs113661867 | T | C | 0.1389 | 0.04085 | 0.0272 | 3.25E-07 |
| Depression | rs116127119 | A | G | 0.2373 | 0.01218 | 0.051 | 3.33E-06 |
| Depression | rs117020343 | G | A | 0.1352 | 0.0422 | 0.0269 | 5.25E-07 |
| Depression | rs12491634 | A | T | 0.7329 | 0.001734 | 0.1595 | 4.34E-06 |
| Depression | rs141654555 | T | C | 0.4577 | 0.003186 | 0.0996 | 4.34E-06 |
| Depression | rs145802336 | A | G | 0.1437 | 0.03772 | 0.0298 | 1.39E-06 |
| Depression | rs189504242 | C | A | 0.1773 | 0.0232 | 0.0373 | 1.95E-06 |
| Depression | rs2424608 | G | C | -0.0718 | 0.8571 | 0.0156 | 4.12E-06 |
| Depression | rs2507900 | A | G | -0.0568 | 0.717 | 0.0121 | 2.54E-06 |
| Depression | rs3135293 | C | T | -0.1359 | 0.04773 | 0.0276 | 8.40E-07 |
| Depression | rs34004743 | A | G | 0.0647 | 0.1809 | 0.0141 | 4.70E-06 |
| Depression | rs367668091 | G | A | 0.182 | 0.02022 | 0.0396 | 4.29E-06 |
| Depression | rs4329857 | T | C | -0.0589 | 0.2411 | 0.0128 | 4.20E-06 |
| Depression | rs4466264 | A | T | 0.0546 | 0.4768 | 0.0109 | 5.97E-07 |
| Depression | rs4880603 | T | G | 0.0559 | 0.2812 | 0.0121 | 4.07E-06 |
| Depression | rs61857747 | A | G | -0.0718 | 0.1426 | 0.0157 | 4.57E-06 |
| Depression | rs7164299 | C | G | 0.0646 | 0.1914 | 0.0139 | 3.21E-06 |
| Depression | rs72662038 | T | C | 0.1158 | 0.05673 | 0.0235 | 8.66E-07 |
| Depression | rs76895136 | G | A | -0.2331 | 0.01453 | 0.047 | 7.04E-07 |
| Depression | rs771122 | C | T | -0.0935 | 0.9062 | 0.0187 | 5.64E-07 |
| Depression | rs8109725 | T | C | -0.0841 | 0.8843 | 0.0172 | 9.94E-07 |
| Depression | rs9371564 | G | A | -0.0576 | 0.6818 | 0.0117 | 8.22E-07 |
| Depression | rs9809577 | A | C | -0.0787 | 0.8233 | 0.0143 | 3.32E-08 |
| Major depressive disorder | rs1021363 | G | A | -0.03 | 0.6434 | 0.0045 | 2.29E-11 |
| Major depressive disorder | rs10235664 | C | T | -0.027 | 0.2529 | 0.0049 | 4.68E-08 |
| Major depressive disorder | rs10913112 | T | C | -0.0262 | 0.378 | 0.0045 | 4.53E-09 |
| Major depressive disorder | rs12919291 | C | G | 0.0327 | 0.1884 | 0.0055 | 3.09E-09 |
| Major depressive disorder | rs12967143 | C | G | -0.0345 | 0.7012 | 0.0047 | 2.53E-13 |
| Major depressive disorder | rs13037326 | T | C | 0.031 | 0.2597 | 0.0049 | 2.40E-10 |
| Major depressive disorder | rs1367635 | C | T | 0.0253 | 0.5148 | 0.0043 | 4.35E-09 |
| Major depressive disorder | rs150186873 | C | A | 0.0704 | 0.0327 | 0.012 | 4.51E-09 |
| Major depressive disorder | rs150346963 | T | C | 0.0283 | 0.4118 | 0.0044 | 1.16E-10 |
| Major depressive disorder | rs17641524 | T | C | -0.03 | 0.2101 | 0.0053 | 1.50E-08 |
| Major depressive disorder | rs1931388 | G | A | -0.0295 | 0.4042 | 0.0044 | 1.68E-11 |
| Major depressive disorder | rs1950829 | G | A | -0.0297 | 0.5173 | 0.0043 | 4.74E-12 |
| Major depressive disorder | rs198457 | T | C | -0.0315 | 0.1886 | 0.0056 | 1.90E-08 |
| Major depressive disorder | rs2111592 | A | G | 0.0263 | 0.3141 | 0.0046 | 1.35E-08 |
| Major depressive disorder | rs2214123 | G | A | -0.0261 | 0.6466 | 0.0045 | 8.56E-09 |
| Major depressive disorder | rs2232423 | G | A | -0.062 | 0.1056 | 0.007 | 1.14E-18 |
| Major depressive disorder | rs2418449 | C | T | -0.0281 | 0.281 | 0.0048 | 4.25E-09 |
| Major depressive disorder | rs247910 | G | A | 0.0237 | 0.457 | 0.0043 | 4.71E-08 |
| Major depressive disorder | rs2522831 | C | T | 0.024 | 0.4739 | 0.0043 | 2.11E-08 |
| Major depressive disorder | rs2568958 | A | G | 0.0382 | 0.6042 | 0.0044 | 2.90E-18 |
| Major depressive disorder | rs28541419 | G | C | -0.0292 | 0.2308 | 0.0052 | 1.76E-08 |
| Major depressive disorder | rs2876520 | G | C | 0.026 | 0.4688 | 0.0043 | 2.24E-09 |
| Major depressive disorder | rs30266 | A | G | 0.0366 | 0.3271 | 0.0046 | 1.43E-15 |
| Major depressive disorder | rs354155 | C | G | -0.0449 | 0.0923 | 0.0075 | 1.75E-09 |
| Major depressive disorder | rs3807865 | A | G | 0.031 | 0.4105 | 0.0044 | 1.09E-12 |
| Major depressive disorder | rs4141983 | C | T | -0.0264 | 0.326 | 0.0046 | 9.69E-09 |
| Major depressive disorder | rs4497414 | C | T | 0.0291 | 0.44 | 0.0044 | 2.93E-11 |
| Major depressive disorder | rs4730387 | A | T | 0.0238 | 0.4659 | 0.0043 | 4.12E-08 |
| Major depressive disorder | rs4799949 | T | C | -0.0292 | 0.6684 | 0.0046 | 1.40E-10 |
| Major depressive disorder | rs4936276 | C | G | 0.0278 | 0.622 | 0.0044 | 3.57E-10 |
| Major depressive disorder | rs508502 | T | C | -0.0264 | 0.2992 | 0.0048 | 3.56E-08 |
| Major depressive disorder | rs59082935 | T | C | 0.0363 | 0.1342 | 0.0066 | 3.07E-08 |
| Major depressive disorder | rs59283172 | A | G | -0.039 | 0.1081 | 0.007 | 2.41E-08 |
| Major depressive disorder | rs61914045 | A | G | 0.0309 | 0.2034 | 0.0054 | 7.96E-09 |
| Major depressive disorder | rs62535714 | A | G | 0.0339 | 0.1639 | 0.0058 | 4.69E-09 |
| Major depressive disorder | rs66511648 | C | T | 0.0297 | 0.284 | 0.0048 | 6.03E-10 |
| Major depressive disorder | rs7152906 | C | T | 0.0258 | 0.5196 | 0.0043 | 1.87E-09 |
| Major depressive disorder | rs7241572 | A | G | 0.0323 | 0.2047 | 0.0054 | 2.43E-09 |
| Major depressive disorder | rs72948506 | A | G | 0.0265 | 0.2975 | 0.0047 | 1.71E-08 |
| Major depressive disorder | rs7538938 | C | T | 0.0251 | 0.5599 | 0.0043 | 7.29E-09 |
| Major depressive disorder | rs754287 | A | T | -0.0289 | 0.3664 | 0.0045 | 1.31E-10 |
| Major depressive disorder | rs7551758 | G | T | 0.0283 | 0.5329 | 0.0043 | 5.11E-11 |
| Major depressive disorder | rs76954012 | A | T | 0.0412 | 0.0931 | 0.0074 | 2.41E-08 |
| Major depressive disorder | rs7725715 | A | G | 0.029 | 0.5343 | 0.0043 | 1.61E-11 |
| Major depressive disorder | rs843812 | A | G | 0.0248 | 0.4117 | 0.0044 | 1.41E-08 |
| Major depressive disorder | rs9364755 | G | A | 0.0283 | 0.2262 | 0.0051 | 3.49E-08 |
| Major depressive disorder | rs9529218 | T | C | -0.034 | 0.2031 | 0.0054 | 2.23E-10 |
| Major depressive disorder | rs9536381 | T | C | 0.0255 | 0.3259 | 0.0046 | 2.62E-08 |
| Major depressive disorder | rs9831648 | T | G | -0.0292 | 0.7739 | 0.0052 | 1.59E-08 |

# EA: effect allele;

# OEA: other-effect allele;

# EAF: effect allele frequency;

#

**Table 2**, Variance explained, F-statistic

|  | **outcome** | **Cervical spondylosis** | **Lumbar disc herniation** | **Rheumatoid arthritis** | **Knee Osteoarthritis** | **Hip Osteoarthritis** | **Osteoporosis** |
| --- | --- | --- | --- | --- | --- | --- | --- |
| **exposure** |  |  |  |  |  |  |  |
| Depression | Variance explained | 1.8% | 1.3% | 3.0% | 3.8% | 3.7% | 4.0% |
|  | F-statistic | 267.8~362.6 | 272.2~312.5 | 267.8~389.3 | 267.8~389.3 | 267.8~389.3 | 267.8~401.8 |
| Major depressive disorder | Variance explained | 1.5% | 1.4% | 1.7% | 1.7% | 1.7% | 1.6% |
|  | F-statistic | 137.8~363.5 | 137.8~349.3 | 137.8~363.5 | 137.8~363.5 | 137.8~363.5 | 137.8~363.5 |

R^2^ was calculated as follows: R^2^ =2×EAF*(1-EAF)*Beta^2^;

The F-statistic for each SNP was calculated as follows: F=(N-2)*R^2^/(1-R^2^).





**Figure 1** (a), Funnel plot of SNP associated with MDD and CS; (b), scatter plot of SNP associated with MDD and CS; (c)Leave-one-out of SNPs associated with MDD and CS.


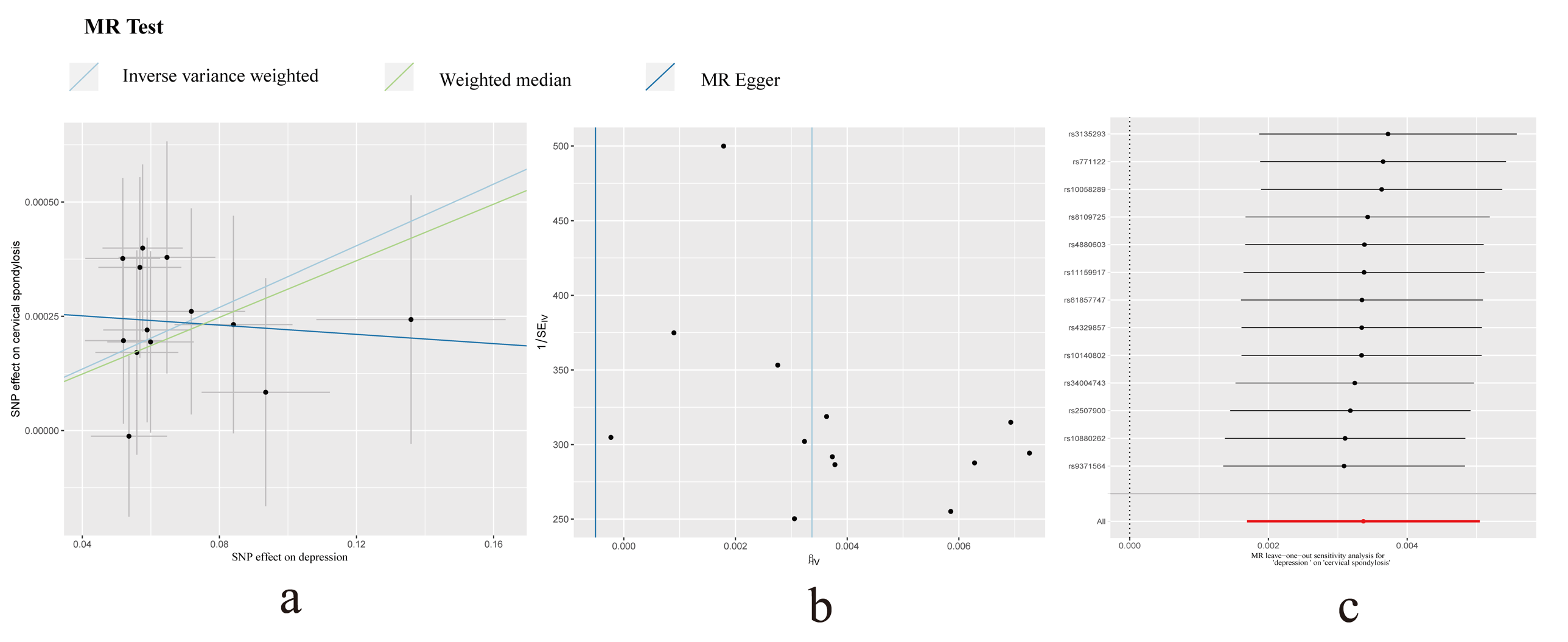


**Figure 2** (a), Funnel plot of SNP associated with Depression and CS; (b), scatter plot of SNP associated with Depression and CS; (c)Leave-one-out of SNPs associated with Depression and CS.





**Figure 3** (a), Funnel plot of SNP associated with MDD and KOA; (b), scatter plot of SNP associated with MDD and KOA; (c)Leave-one-out of SNPs associated with MDD and KOA.
